# Pretransplant malnutrition, inflammation, and atherosclerosis affect cardiovascular outcomes after kidney transplantation

**DOI:** 10.1186/s12882-015-0108-3

**Published:** 2015-07-21

**Authors:** Jin Ho Hwang, Jiwon Ryu, Jung Nam An, Clara Tammy Kim, Hyosang Kim, Jaeseok Yang, Jongwon Ha, Dong Wan Chae, Curie Ahn, In Mok Jung, Yun Kyu Oh, Chun Soo Lim, Duck-Jong Han, Su-Kil Park, Yon Su Kim, Young Hoon Kim, Jung Pyo Lee

**Affiliations:** Department of Internal Medicine, Chung-Ang University Hospital, Seoul, South Korea; Department of Internal Medicine, Hallym University College of Medicine, Chuncheon, South Korea; Department of Internal Medicine, Seoul National University Boramae Medical Center, Seoul, South Korea; Graduate School of Public Health, Seoul National University, Seoul, South Korea; Department of Internal Medicine, Asan Medical Center and University of Ulsan College of Medicine, Seoul, South Korea; Transplantation Center, Seoul National University Hospital, Seoul, South Korea; Department of Internal Medicine, Seoul National University Bundang Hospital, Seongnam, South Korea; Department of Internal Medicine, Seoul National University College of Medicine, Seoul, South Korea; Department of Surgery, Seoul National University Boramae Medical Center, Seoul, South Korea; Department of Surgery, Asan Medical Center and University of Ulsan College of Medicine, Seoul, South Korea

**Keywords:** Acute Coronary Syndrome, Atherosclerosis, Cardiovascular Outcome, Inflammation, Kidney Transplantation, Malnutrition

## Abstract

**Background:**

Malnutrition, inflammation, and atherosclerosis (MIA) syndrome is associated with a high mortality rate in patients with end-stage renal disease. However, the clinical relevance of MIA syndrome in kidney transplantation (KT) recipients remains unknown.

**Methods:**

We enrolled 1348 adult KT recipients. Recipients were assessed based on serum albumin, cholesterol, or body mass index for the malnutrition factor and C-reactive protein level for the inflammation factor. Any history of cardiovascular (CV), cerebrovascular, or peripheral vascular disease satisfied the atherosclerosis factor. Each MIA factors were assessed by univariate analysis and we calculated an overall risk score by summing up scores for each independent variable. The enrolled patients were divided into 4 groups depending on the MIA score (0, 2–4, 6, 8–10).

**Results:**

The patients with higher MIA score showed worse outcome of fatal/non-fatal acute coronary syndrome (ACS) (*p* < 0.001) and composite outcomes of ACS and all-cause mortality (*p* < 0.001) than with the lower MIA score. In multivariate analysis, ACS showed significantly higher incidence in the MIA score 8-10 group than in the MIA score 0 group (Hazard ratio 6.12 95 % Confidence interval 1.84–20.32 *p* = 0.003).

**Conclusions:**

The presence of MIA factors before KT is an independent predictor of post-transplant CV outcomes.

## Background

Cardiovascular disease (CVD) is the main cause of morbidity and mortality in patients with end-stage renal disease (ESRD) despite major research efforts and improvements in dialysis technology [1]. There have been many discussions about traditional risk factors that may not sufficiently predict CVD occurrence in patients with ESRD [2–5].

Malnutrition, inflammation, and atherosclerosis (MIA) syndrome is associated with a high mortality rate and increased cardiovascular event rate in patients with ESRD [6]. The 3 factors of MIA syndrome interact with each other and create a vicious cycle [7]. Malnutrition or protein-energy wasting may aggravate existing inflammation, accelerating atherosclerosis and increasing susceptibility to infection [8, 9]. Chronic inflammation is common in patients with chronic kidney disease, in part because of the decreased glomerular filtration rate (GFR), and also because of the dialysis procedure [10, 11]. Inflammation plays a key role in atherosclerosis and may contribute to an increased cardiovascular mortality associated with endothelial dysfunction and increased oxidative stress [9, 12]. With these observations, the term malnutrition inflammation complex syndrome (MICS) was coined.

Kidney transplantation provides a better quality of life for patients with ESRD. With the development and advancement of immunosuppressive agents, renal allograft survival rates have improved over the years. Even after the transplantation, CVD is an important cause of death [13]. Moreover, the immunosuppressive agents increase atherosclerotic risk by elevating blood pressure and by aggravating several metabolic profiles such as dyslipidemia and new onset diabetes after transplantation (NODAT) [13, 14].

While several studies about MICS or MIA syndrome in patients with ESRD have been reported [6, 7, 9, 15], only a few reports have evaluated transplant wait-listed patients with ESRD and the association between pretransplant parameters (e.g., C-reactive protein (CRP), albumin) and post-transplant outcomes [16–18]. Thus, the clinical relevance of MIA syndrome in kidney transplant recipients remains unclear. We hypothesized that MIA syndrome is associated with poorer post-transplant outcome.

## Methods

### Ethics statement

This study was approved by the institutional review board at Seoul National University Hospital (H-1302-018-462), and the need for informed consent from the patients was waived because of the retrospective study design. All clinical investigations were conducted in accordance with the guidelines of the 2008 Declaration of Helsinki.

### Study design and patients

**Fig. 1:**
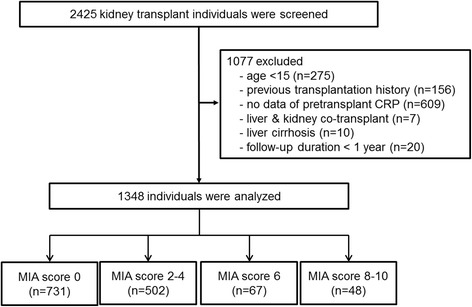
Defining the study population. We reviewed the medical records of 2425 individuals and collected data from 1348 patients

This study was performed as a retrospective, multicenter study. Among the patients in whom kidney transplantation had been performed at Seoul National University Hospital, Seoul National University Boramae Medical Center, Seoul National University Bundang Hospital, and Asan Medical Center from Jun. 1999 through Dec. 2011, we reviewed the medical records of 2425 individuals and collected data from 1348 patients. All patients were adults (age ≥ 15 years); had pretransplant CRP, serum albumin, and cholesterol data available; received renal transplants; and were followed for more than one year after transplantation (Fig. 1). Patients with a previous transplantation history and those with unavailable pretransplant laboratory profiles were excluded. Patients with a follow-up duration less than one year were also excluded from the analysis.

Clinical parameters such as age at the time of kidney transplantation, sex, body mass index (BMI), laboratory test results (CRP, serum albumin, cholesterol, intact patathyroid hormone (PTH), and cytomegalovirus (CMV) immunoglobulin G (IgG) status), underlying diseases (hypertension, diabetes mellitus (DM), ischemic heart disease, liver cirrhosis, and tuberculosis), dialysis modality, and duration of dialysis before transplantation were collected. Donor factors such as age at transplantation, sex, donor source, and CMV IgG status were evaluated.

### Definitions and grouping

We evaluated enrolled patients by defining and calculating MIA factors. Between patients with or without acute coronary syndrome (ACS), the univariate Cox regression was performed to identify the influence of variables such as serum albumin, cholesterol, and BMI for malnutrition factor, and CRP for inflammation factor. Researchers considered the atherosclerosis factor to be satisfied if there was any history of CV, cerebrovascular, or peripheral vascular disease. Recipients were assessed according to the quartile value of pretransplant albumin and CRP. We evaluated each independent variable for the assumption of proportional hazards. From the regression model, the β-coefficient of each variable was assigned score by rounding to the nearest integer [19]. After that, we calculated an overall risk score by summing up the scores for each independent predictor variable for a given patient. Subsequently, the enrolled patients were divided into four groups depending on the MIA score considering score homogeneity (0, 2–4, 6, 8–10).

Researchers have checked cholesterol, albumin, and CRP levels at least two time points within one year before transplantation and used mean value of the each variables. The minimum interval between the two time points was four weeks.

The cardiovascular outcome of fatal/non-fatal ACS was defined as usual ACS, which includes unstable angina/non-ST elevated myocardial infarction (MI) and ST elevated MI. In this study all the patients with ACS were confirmed by coronary angiography.

### Primary and secondary objectives

The primary objective of the study was to evaluate whether pretransplant MIA factors affect the occurrence of fatal/non-fatal ACS after transplantation. The secondary objective of the study was to evaluate the composite of ACS with all-cause mortality and graft failure. Graft failure was defined as a requirement for maintenance dialysis or re-transplantation.

### Statistical analysis

All analyses and calculations were performed by using the IBM SPSS Statistics V21.0 (IBM Corporation, Armonk, NY, USA). Continuous variables were expressed as the mean ± standard deviation and as the percentage for categorical variables. Continuous and categorical data were compared by using the Student’s *t-*test and the chi-square test, respectively. The ACS-free survival rates and other event-free survival rates were calculated by using the Kaplan-Meier method, and comparison between groups was performed by using the log-rank test. The Cox regression model was used to identify risk factors and to calculate the hazard ratio (HR) and 95 % confidence interval (CI). The corresponding β-coefficient for each independent variable from the regression model for the occurrence of ACS was used to calculate MIA score. Differences with *p* < 0.05 were considered statistically significant.

## Results

### Baseline characteristics of enrolled patients

The baseline characteristics of the 1348 enrolled patients are described in Table 1. The mean age of patients at transplantation was 43.0 years, and 57.2 % were men (*n* = 771). The mean follow-up duration was 57.0 ± 36.0 months. The pretransplant BMI, albumin, CRP, and cholesterol levels were 22.4 ± 3.2 kg/m^2^, 3.7 ± 0.5 g/dL, 0.48 ± 1.17 mg/dL, and 158.8 ± 40.9 mg/dL, respectively. Hypertension was diagnosed in 1198 patients (88.9 %) and DM was diagnosed in 275 patients (20.4 %). One-hundred fifteen patients (8.5 %) had experienced at least one of the following vascular diseases: CVD, cerebrovascular disease, or peripheral vascular disease. There were more patients with dyslipidemia in the higher MIA score group (63.8 % in MIA score 8-10 group, 26.6 % in average). There was no significant difference in total cholesterol level between the patients who took the lipid lowering agent and the patients who did not (156.9 ± 45.8 vs. 159.4 ± 38.8, *p* = 0.38, data not shown). Preemptive kidney transplantation was performed in 217 patients (16.1 %), and hemodialysis was performed frequently before transplantation (*n* = 679, 60.0 % of dialysis patients). Most enrolled patients received only kidney transplantation (*n* = 1211, 89.8 %), and most grafts were received from living donors (*n* = 1065, 79.0 %). The prescription of immunosuppressive agents after transplant was not different between the groups.

**Table 1:** Baseline characteristics of the study subjects

	MIA score 0	MIA score 2–4	MIA score 6	MIA score 8–10	Total	p-value
	(*n* = 731)	(*n* = 502)	(*n* = 67)	(*n* = 48)	(*N* = 1348)	
Age at the transplantation^a^	41.1 ± 11.9	42.8 ± 12.0	52.5 ± 10.4	57.7 ± 8.0	43.0 ± 12.3	<0.001^‡^
Recipient’s sex (male, %)	54.7	57.4	67.2	79.2	57.2	0.003
Body weight (kg)^a^	59.9 ± 10.7	61.4 ± 12.0	62.4 ± 11.0	64.8 ± 9.0	60.8 ± 11.2	0.003^‡^
Height (cm)^a^	164.2 ± 8.6	164.4 ± 8.8	164.4 ± 7.8	166.4 ± 7.4	164.4 ± 8.6	0.374
BMI (kg/m^2^)^a^	22.1 ± 2.9	22.6 ± 3.5	23.0 ± 3.3	23.3 ± 2.6	22.4 ± 3.2	0.001^§^
Current smoker (%)	10.6	9.1	23.3	6.4	9.8	<0.001
Past medical history						
Hypertension (%)	88.5	87.3	100.0	95.8	88.9	0.007
DM (%)	14.1	21.5	49.3	64.6	20.4	<0.001
Vascular diseases (%)^b^	0.0	0.0	100.0	100.0	8.5	<0.001
Dyslipidemia (%)	21.4	29.1	35.0	63.8	26.6	<0.001
Renal disease causing ESRD						0.033
DM (%)	17.4	19.4	14.3	12.2	17.8	
Hypertension (%)	10.0	9.7	17.9	12.2	10.4	
Glomerulonephritis (%)	30.5	28.4	21.4	22.0	29.0	
PKD (%)	5.3	3.9	14.3	4.9	5.2	
Others (%)	36.8	38.6	32.1	48.7	37.6	
Laboratory Findings						
Serum albumin (g/dl)^a^	3.9 ± 0.4	3.4 ± 0.5	3.9 ± 0.3	3.3 ± 0.6	3.7 ± 0.5	<0.001^‡§^
CRP (mg/dl)^a^	0.11 ± 0.11	0.97 ± 1.62	0.15 ± 0.13	1.25 ± 2.46	0.48 ± 1.17	<0.001^‡§^
Total cholesterol (mg/dl)^a^	161.6 ± 37.5	155.7 ± 44.9	156.5 ± 38.4	151.6 ± 48.5	158.8 ± 40.9	0.044
Triglyceride (mg/dl)^a^	120.8 ± 67.2	125.9 ± 99.2	135.6 ± 89.5	133.6 ± 72.9	123.9 ± 81.1	0.419
LDL cholesterol (mg/dl)^a^	91.5 ± 32.1	91.3 ± 31.8	87.2 ± 31.6	93.6 ± 43.2	91.3 ± 32.4	0.772
Intact PTH (pg/ml)^a^	262.2 ± 312.8	238.7 ± 242.7	181.8 ± 183.8	249.7 ± 273.3	249.6 ± 279.4	0.29
CMV IgG positive (%)	93.1	96.0	92.9	96.3	94.8	0.475
Dialysis before TPL (%)	82.2	85.7	88.1	85.4	83.9	0.491
Preemptive TPL (%)	17.8	14.3	11.9	14.6	16.1	0.491
HD:PD:both or switch (%)	60.7:18.4:3.1	57.8:24.0:3.9	63.3:18.3:6.5	68.8:14.6:2.0	60.0:20.4:3.5	0.308
Dialysis duration (months)^a^	26.7 ± 38.8	27.7 ± 38.0	38.4 ± 50.8	21.2 ± 27.7	27.4 ± 38.8	0.098
Donor information						
Deceased donor (%)	20.1	21.3	29.9	17.0	21.0	0.003
Donor’s age (year)^a^	40.2 ± 11.9	40.0 ± 12.4	41.7 ± 13.3	43.0 ± 12.8	40.3 ± 12.2	0.303
Donor’s sex (male, %)	52.5	57.7	50.0	62.5	54.6	0.178
CMV IgG positive (%)	54.9	69.9	47.8	60.4	60.3	<0.001^§^
TPL (KT only : SPK : Others, %)	89.1:3.1:7.8	91.4:4.8:3.8	85.1:4.5:10.4	89.6:2.1:8.3	89.8:3.8:6.4	0.053
Immunosuppressive agents						
Steroid maintenance strategy (%)	92.6	89.5	100	85.7	90.9	0.625
CNI (CsA : Tacrolimus, %)	34.2:65.3	35.8:63.6	22.8:75.4	29.7:70.3	34.0:65.4	0.254
Antimetabolites (Aza : MMF, %)	6.5:88.1	10.2:79.4	10.5:80.7	8.6:82.9	8.0:84.6	0.095

*BMI* body mass index, *DM* diabetes mellitus, *ESRD* end-stage renal disease, *PKD* polycystic kidney disease, *CRP* c-reactive protein, *LDL* low-density lipoprotein, *TPL* transplantation, *HD* hemodialysis, *PD* peritoneal dialysis, *KT* kidney transplantation, *SPK* simultaneous pancreas-kidney transplant, *CNI* calcineurin inhibitor, *CsA* cyclosporine A, *Aza* azathioprine, *MMF* mycophenolate mofetil

^a^data are expressed as the mean ± SD

^b^Vascular disease included cardiovascular, cerebrovascular, and peripheral vessel diseases

^‡^ *p* < 0.05 at post-hoc analysis between MIA score 0 group and MIA score 8–10 group

^§^ *p* < 0.05 at post-hoc analysis between MIA score 6 group and MIA score 8–10 group

### Pretransplant MIA factor profile

The HR for cardiovascular disease of albumin, CRP, and previous history of vascular disease was 2.040, 1.893, and 6.41 respectively. The corresponding scores for the same MIA factors were 2, 2, and 6 (Table 2). Seven-hundred thirty-one patients (54.2 %) did not satisfy any of the MIA factors (MIA score 0). Three-hundred ninety-eight patients (29.5 %) had one MIA factor with score 2 and 67 patients (5.0 %) had 1 MIA factor with score 6. Nine patients had all of three MIA factors (score 10). Other MIA score groups and each component are shown in Table 3. The distribution of MIA scores of the patients with pre-emptive transplantation was not different with the patients who underwent dialysis before transplantation (*p* = 0.451, data not shown).

**Table 2:** Factors associated with the occurrence of ACS

	Univariate			Multivariate^a^		
	HR	95 % CI	p-value	HR	95 % CI	p-value
Age	1.08	1.03–1.12	0.001	1.05	1.01–1.10	0.008
Male	3.54	1.45–8.60	0.005	2.26	0.89–5.71	0.085
Smoking	1.92	0.77–4.78	0.162			
BMI (continuous variable)	1.11	1.00–1.23	0.049			
Hypertension	4.27	0.58–31.44	0.154	-	-	-
DM	3.55	1.78–7.05	<0.001	2.58	0.99–6.76	0.054
Dialysis before KT (vs. preemptive)	1.58	0.46–5.38	0.469	-	-	-
Dialysis vintage (months)	1.01	0.99–1.013	0.146			
Cholesterol (<150 mg/dL)	1.46	0.69–3.07	0.324			
Intact PTH	1.00	0.99–1.00	0.384			
Recipient CMV IgG (+)	0.01	0.01–20.16	0.998			
Donor sex (male)	0.64	0.31–1.29	0.208	-	-	-
Donor age	1.03	0.99–1.06	0.094	-	-	-
Deceased donor (vs. living donor)	2.72	1.12–6.61	0.027	2.17	0.80–5.91	0.128
Donor CMV IgG (+)	0.891	0.44–1.79	0.745			
CsA (vs. tacrolimus)	1.9	0.91–3.95	0.085	-	-	-
NODAT	2.80	1.24–6.32	0.013	2.88	1.03–8.10	0.045
CMV disease	0.046	0.01–14.33	0.686	0.15	0.01–41.48	0.712
MIA factors						
Albumin (lowest quartile vs. others)	2.04	0.98–4.24	0.056	2.33	1.11–4.88	0.026
CRP (highest quartile vs. others)	1.89	0.94–3.82	0.075	1.55	0.75–3.20	0.237
Previous history of vascular disease^a^	6.41	3.09–13.33	<0.001	2.61	1.08–6.27	0.033
MIA score^a^						
0	Reference			Reference		
2	1.17	0.45–3.05	0.742	2.14	0.77–5.96	0.147
4	2.51	0.79–8.03	0.121	1.86	0.39–8.73	0.436
6	5.23	1.76–15.53	0.003	2.93	0.90–9.54	0.075
8	7.53	2.28–24.85	0.001	3.11	0.73–13.29	0.125
10	32.96	7.30–148.94	<0.001	34.29	8.29–141.84	<0.001
Group by MIA score^a^						
0	Reference			Reference		
2–4	1.33	0.56–3.16	0.517	2.05	0.79–5.35	0.142
6	5.28	1.78–15.68	0.003	3.06	0.94–9.99	0.063
8–10	11.18	4.12–30.33	<0.001	6.12	1.84–20.32	0.003

Data were analyzed by using the Cox regression, Backward LR method in the multivariate analysis

*ACS* acute coronary syndrome, *DM* diabetes mellitus, *KT* kidney transplantation, *NODAT* new onset diabetes after transplantation, *CsA* cyclosporine A, *Alb* albumin

^a^We performed multivariate analysis about “MIA score”, “Previous history of vascular disease”, and “Group by MIA score” separately with other variables

**Table 3:** Crosstable by MIA score group and each component

	MIA score 0	MIA score 2	MIA score 4	MIA score 6	MIA score 8	MIA score 10	Total
	(*n* = 731)	(*n* = 398)	(*n* = 104)	(*n* = 67)	(*n* = 39)	(*n* = 9)	(*N* = 1348)
Albumin lower quartile (%)^a^	0	191 (48.0)	104 (100)	0	24 (61.5)	9 (100)	335 (24.3)
CRP upper quartile (%)^a^	0	207 (52.0)	104 (100)	0	15 (38.5)	9 (100)	340 (24.9)
Atherosclerosis (%)^a^	0	0	0	67 (100)	39 (100)	9 (100)	116 (8.5)

^a^Data are expressed as patients number (% by MIA score group).

### Factors related to the occurrence of ACS

Univariate analysis of the association between ACS and covariates showed a significant relationship with age (HR, 1.08; 95 % CI, 1.03–1.12; *p* = 0.001); male sex (HR, 3.54; 95 % CI, 1.45–8.60; *p* = 0.005); BMI (HR, 1.11; 95 % CI, 1.00–1.23; *p* = 0.049); DM (HR, 3.55; 95 % CI, 1.78–7.05; *p* < 0.001); deceased donor (HR, 2.72; 95 % CI, 1.12–6.61; *p* = 0.027); NODAT (HR, 2.80; 95 % CI, 1.24–6.32; *p* = 0.013); previous history of vascular disease (HR, 6.41; 95 % CI, 3.09–13.33; *p* < 0.001); and the MIA score group (10 vs. 0; HR, 32.96; 95 % CI, 7.30–148.94; *p* < 0.001 and 8–10 vs. 0; HR, 11.18; 95 % CI, 4.12–30.33; *p* < 0.001). Of these, MIA score 8–10 (HR, 6.12; 95 % CI, 1.84–20.32; *p* = 0.003); age (HR, 1.05; 95 % CI, 1.01–1.10; *p* = 0.008); NODAT (HR, 2.88; 95 % CI, 1.03–8.10; *p* = 0.045); albumin (HR, 2.33; 95 % CI, 1.11–4.88; *p* = 0.026); and previous history of vascular disease (HR, 2.61; 95 % CI, 1.08–6.27; *p* = 0.033) remained significant predictors on multivariate analysis (Table 2). There was no association between ACS and smoking history, hypertension, cholesterol level, intact PTH level, recipient and donor CMV IgG antibody status, donor sex, donor age, dialysis modality before transplantation, immunosuppressive agents, or CMV disease.

### ACS based on each MIA factor and impact of ACS on mortality

**Fig. 2:**
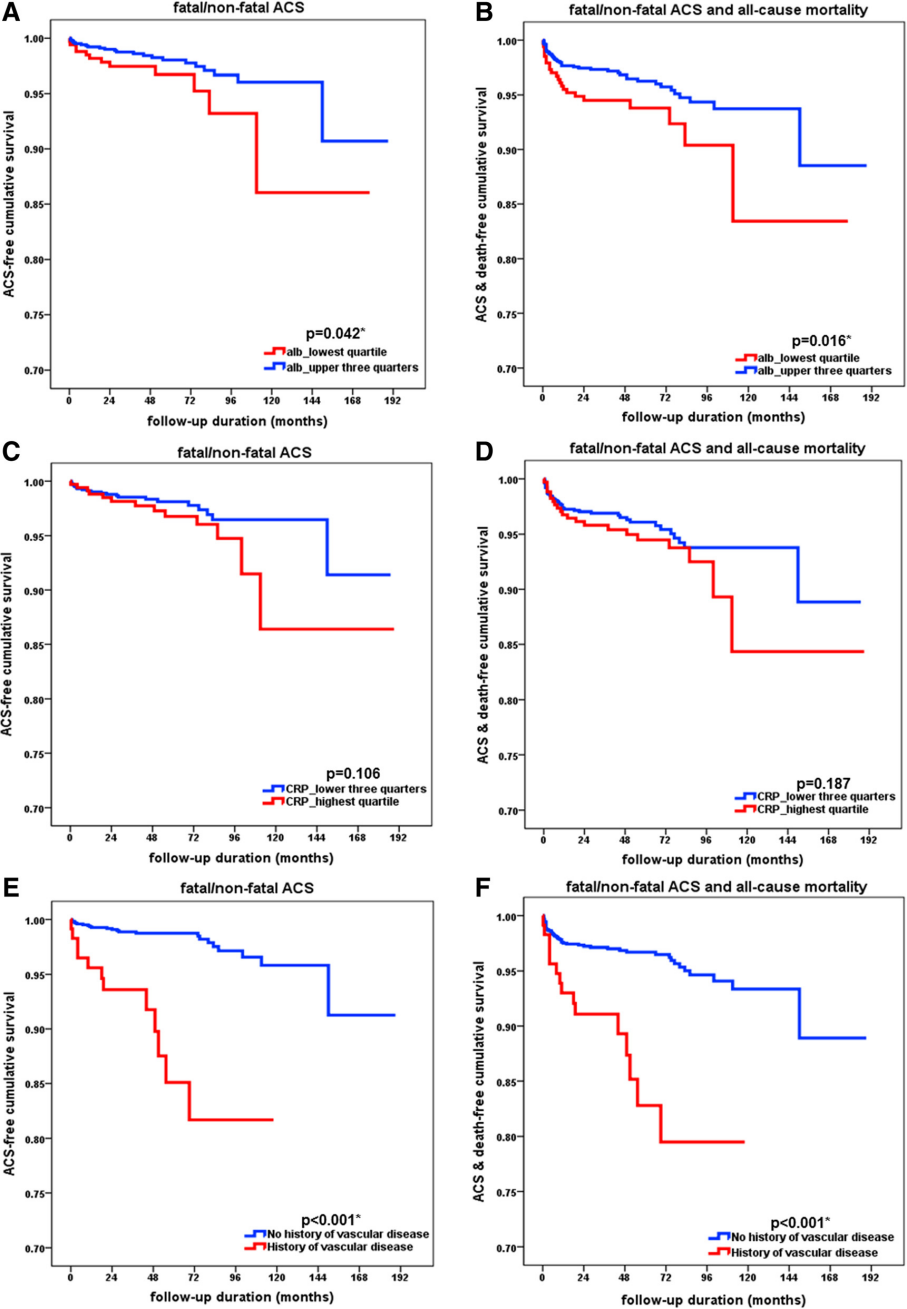
Kaplan-Meier curves of fatal/non-fatal acute coronary syndrome (ACS) and composite outcomes of ACS and death in patients with different MIA factors. **a**-**b**. The patients in the lowest albumin quartile showed worse ACS outcomes (*p* = 0.042) and composite outcomes of ACS and death (*p* = 0.016) than the others. **c**-**d**. The patients in the highest CRP quartile showed tendency of worse ACS outcomes (*p* = 0.106) and composite outcomes of ACS and death (*p* = 0.187) although statistically insignificant. **e**-**f**. The patients with previous history of vascular disease showed poor outcome of ACS (*p* < 0.001) and composite outcomes of ACS and death (*p* < 0.001) than the others

There was a significant difference in occurrence of ACS (*p* = 0.042) and composite outcomes (*p* = 0.016) between the lowest quartile and the others when albumin level was analyzed (Fig. 2a, b). Our findings indicate that increased CRP level was not predictive of ACS (*p* = 0.106) or composite outcomes (*p* = 0.187) (Fig. 2c, d). Previous history of vascular disease (any cardiovascular, cerebrovascular, or peripheral vascular disease) was significantly related with ACS (*p* < 0.001) and composite outcomes (*p* < 0.001) (Fig. 2e, F). In 1233 patients without a history of vascular disease, 21 patients experienced ACS after transplantation. However, in 115 patients with a history of vascular disease, 12 patients experienced ACS.

The all-cause mortality was significantly higher in the patients with post-transplant CV events than in the other patients (21.2 % vs. 2.5 %; *p* < 0.001). Cardiovascular events were the second most common cause of post-transplant mortality in this study population.

### MIA score groups and occurrence of ACS

**Fig. 3:**
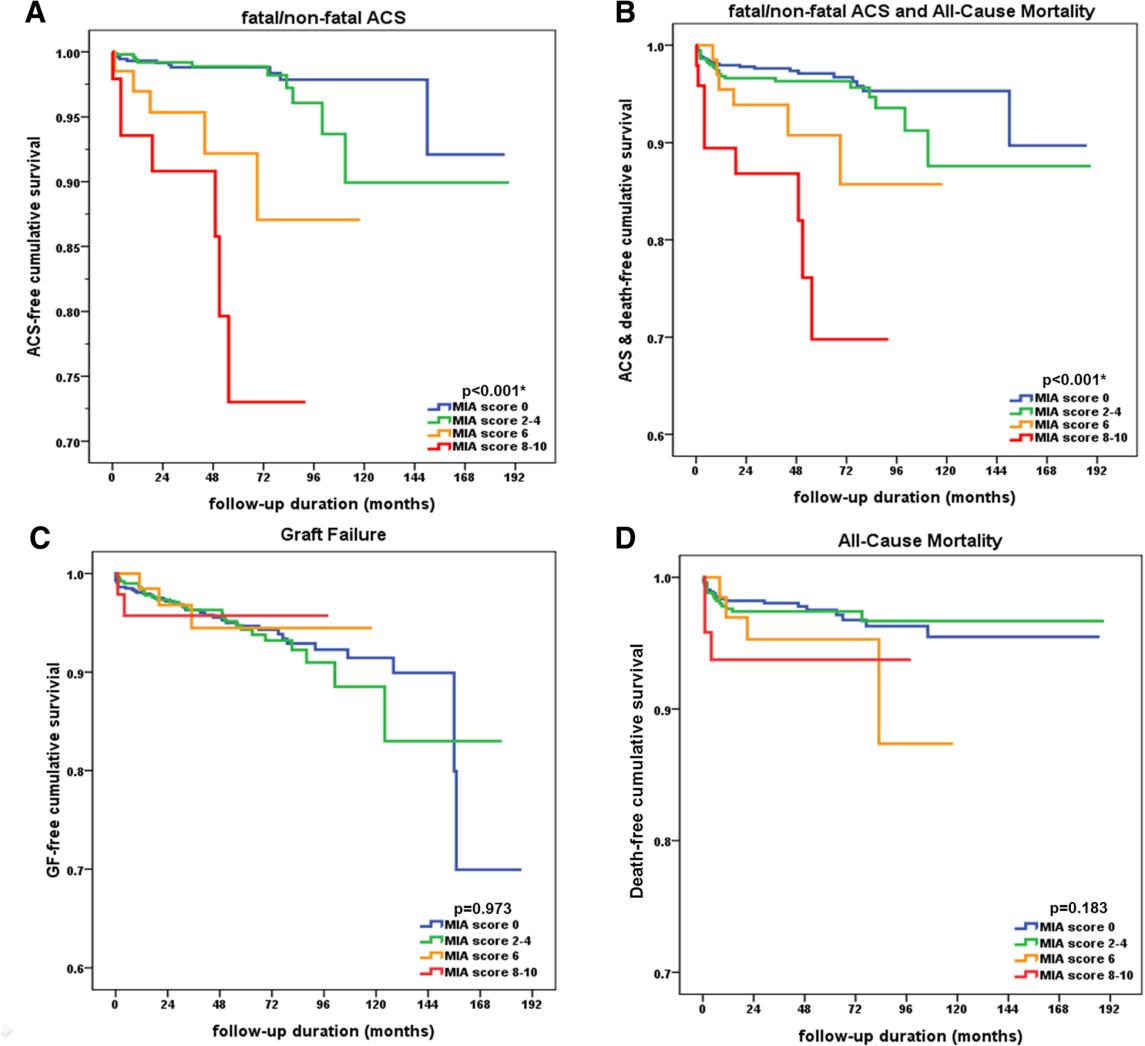
Kaplan-Meier curves of ACS, composite outcomes of ACS, graft outcome, and death in patients in MIA score groups. **a**-**b**. The patients with higher MIA score showed worse outcome of ACS (*p* < 0.001) and composite outcomes of ACS and death (*p* < 0.001) than those with lower MIA scores. **c**-**d**. Graft outcome and all-cause mortality were not different between the groups (*p* = 0.973 and *p* = 0.183, respectively)

The patients were divided into 4 groups depending on MIA scores and the ACS, graft outcome, and mortality were evaluated between groups. The ACS occurred in total of 33 patients (2.4 %). The patients with higher MIA scores showed worse ACS outcomes (*p* < 0.001, Fig. 3a) and composite outcomes of ACS and death (*p* < 0.001, Fig. 3b) than those in the group with lower MIA scores.

**Fig. 4:**
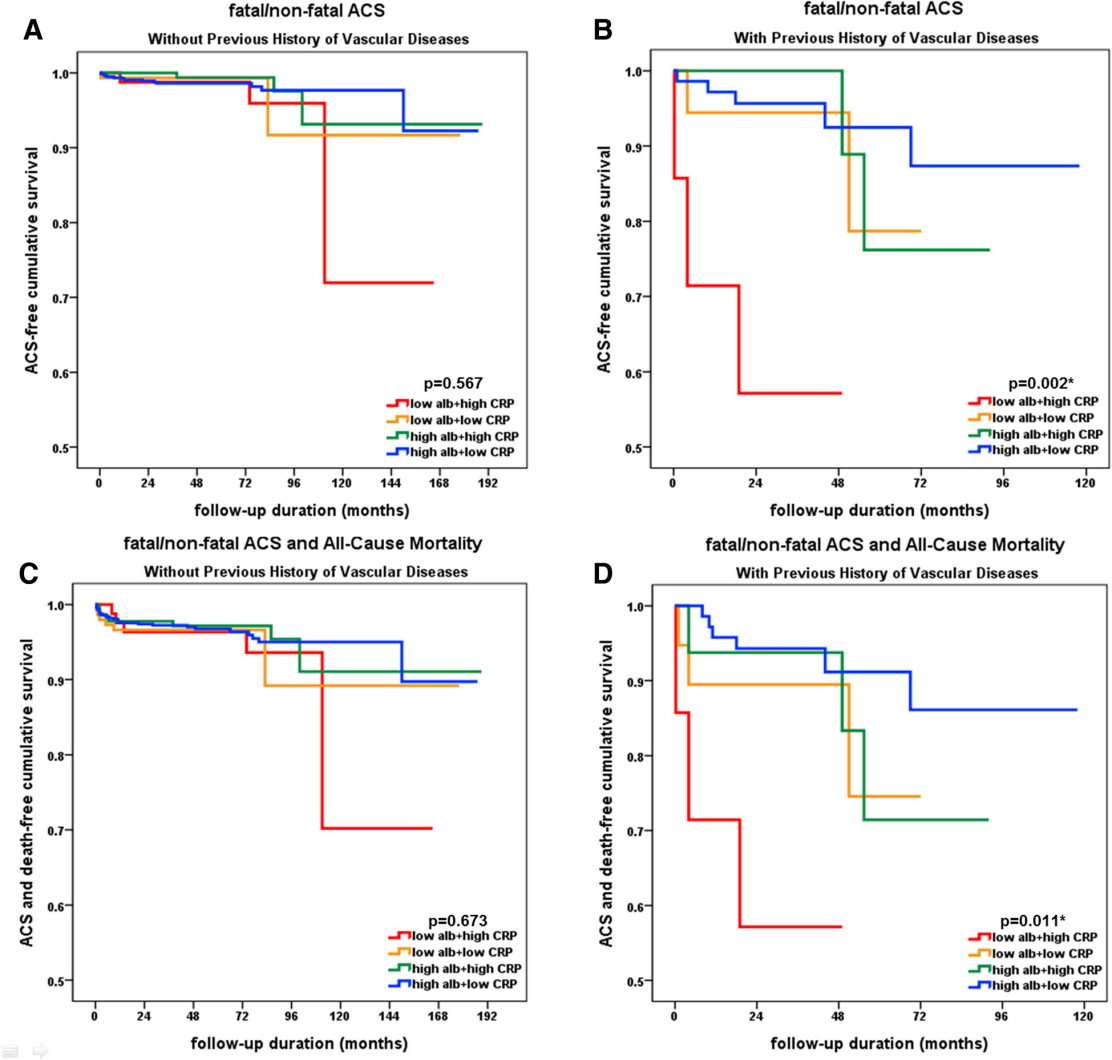
Kaplan-Meier curves of ACS and composite outcomes of ACS and death in divided patients by previous history of vascular disease. **a**-**c**. In the patients without a history of vascular disease, ACS (*p* = 0.567) and composite outcome (*p* = 0.673) did not show significant difference according to the quartile value of albumin and CRP. **b**-**d**. In the patients with a history of vascular disease, ACS (*p* = 0.002) and composite outcome (*p* = 0.011) occurred more frequently in the patients in the lowest albumin + highest CRP quartile group than in the other patients

To eliminate the strong effect of vascular disease history, we divided the patients by history of vascular disease and evaluated separately. In patients without previous vascular disease, ACS and composite outcome did not show significant difference according to the quartile value of albumin and CRP (Fig. 4a, c). However, in the patients with a history of vascular disease, ACS (*p* = 0.002, Fig. 4b) and composite outcome (*p* = 0.011, Fig. 4d) occurred more in the lowest albumin + highest CRP quartile group than in the other groups.

### Other outcomes based on MIA factor

The overall survival rate was 97.0 %, and all-cause mortality rate between the groups did not differ based on MIA score. Infection (23 patients, 56.1 %) and cardiovascular disease (5 patients, 12.2 %) were the most common causes of death after transplantation. Eight patients (19.5 %) died suddenly from unknown cause.

During a mean follow-up duration of 57.0 ± 36.0 months, the overall graft survival rate was 94.6 %, excluding patients who died with functioning grafts. Graft outcome and all-cause mortality were not different between the groups (*p* = 0.973 and *p* = 0.183) (Fig. 3c, d). Acute rejection and acute kidney injury related with sepsis was the first and the second most common cause of graft failure (23.3 % and 13.7 %, respectively). Transplant glomerulopathy (9.6 %), recurred glomerulonephritis (6.8 %), non-compliance to immunosuppressive agents (6.8 %), BK virus-induced nephropathy (5.5 %), and renal infarct (4.1 %) were the next common causes. The number of total and graft failure cases in MIA score 6 and 8-10 groups were not enough to perform statistical analysis on the graft failure causes.

The occurrence of CMV disease after KT was not different between the groups, which could be a strong predictor of cardiovascular complications (7.7 %, 4.3 %, 7.1 %, and 3.7 % in MIA score 0, 2–4, 6, and 8–10; *p* = 0.359).

## Discussion

In this study of 1348 kidney transplant recipients, we report independent associations of pretransplant MIA score with the occurrence of post-transplant ACS. To date, few reports have examined the association between pretransplant MICS and post-transplant outcomes. Most of these reports focused on different outcomes such as mortality (including cardiovascular mortality and all-cause mortality), delayed graft function, chronic allograft nephropathy, or graft outcomes [17, 20–23]. Some were either small in sample size [18] or focused on association only with one MIA factor (albumin or CRP level) [24]. To our knowledge, this is the first multicenter study on the occurrence of ACS in kidney transplant recipients with combined factor for considering pretransplant MIA score.

We defined malnutrition and inflammation arbitrarily in some ways. However, an albumin level < 3.5 mg/dL was not only a lower quartile value, but also “below normal level”. Also a cholesterol level < 150 mg/dL originated with the malnutrition standards of dialysis patients [25]. In 2003, the American Heart Association and the Centers for Disease Control and Prevention endorsed cutoff points of < 1 mg/dL for low-risk CRP in the adult nontransplanted population, but we applied the lower cutoff point (>0.5 mg/dL) for CRP as we used higher quartile of enrolled populations [26].

Interest in CV outcomes after kidney transplantation is likely to increase because long-term graft outcomes have improved. The current study revealed that ACS were significantly high in the higher MIA score group compared to the lower MIA score group after multivariate analysis by adjusting possible factors with association. Increasing patient age, DM, male sex, hypertension, dyslipidemia, and cigarette smoking are well known risk factors for CV disease in general [27]. Non-traditional risk factors such as dialysis vintage before transplantation, rejection, anemia, proteinuria, reduced kidney function following transplantation, and elevated inflammatory markers have been associated with increased CV risk in various studies [28, 29]. Pretransplant CV disease is the most important predictor of post-transplant CV events [30]. Further, both pretransplant diabetes and NODAT are associated with the occurrence of post-transplant CV outcomes [31]. Our results are meaningful because we showed the independent association between the pretransplant MIA score group with ACS after all known risk factors were adjusted.

Our data showed significant result only in ACS and composite outcome. Graft function and other risk factors are known to be associated with cardiovascular outcome [32]. However, in this study, graft failure did not affect the occurrence of fatal/non-fatal ACS.

In this study, pretransplant CRP level did not show independent association with post-transplant ACS, although several former studies reported an association between high CRP levels and CV outcomes [18, 24, 29]. The occurrence of ACS in the patients with sustained high CRP levels after transplantation cannot be verified, because post-transplant one-year CRP value was not measured routinely in all patients.

The overall mortality rate was only 3.0 % in this study. The population was too small to analyze statistical significance and to affect the composite outcome with ACS. For the other aspect, patients with a history of vascular disease are usually selected more carefully before entering the waiting list for KT. This could be another reason why the mortality rate was not different with others. Graft failure also failed to show a difference between groups.

The nutritional status of Korean might be different with a Western population and the risk of coronary heart disease is lower in Korean population [33]. The CVD is not the primary cause of death even though the mortality of unknown cause (19.5 %) was assumed as CV death. To the best of our knowledge, our study is notable for enrolling the largest number of Korean patients in multicenter to provide the analysis for the correlation between the pretransplant MIA factors and ACS. Enrolled clinical institutions are active centers for transplantation in South Korea. We evaluated patients with relatively simple standards that can be easily applied. Also, we used cardiovascular outcome as “ACS”, which is specific outcome of ischemic heart disease. The term ‘major adverse cardiac event’ was commonly used for several studies, but it is considered as inconsistent and of heterogeneous definition [34].

The following limitations should be considered when interpreting our results. Like all other retrospective studies, this study cannot prove a causal relationship. There could be inevitable biases which are patient selection, follow-up, or interpretation of the outcomes. However, we tried to minimize the possible biases by considerate medical record review and by including every factor which can possibly affect the outcome of patients. Also, it was not able to perform the true assessment of nutrition such as subjective global assessment of nutritional status. The method used to calculate the MIA score might need further validation even which was published previously in other fields [19]. The CRP was not a routine laboratory test before and after transplantation in many clinical institutions, and patients who did not have pretransplant serum CRP levels measured were excluded from analysis. Moreover, transferrin is a known marker of malnutrition in patients with ESRD [35], but we could not evaluate transferrin as a malnutrition marker. As the population of this study is all Asian, the results are not directly comparable with US or European cohorts. The heterogeneity of the populations (transplantation types and preemptive KT vs. dialysis before KT) can be the other limitation of this study although we verified such clinical findings were not different between the MIA score groups. Lastly, we applied our own standard for classification because there is no standard method to assess malnutrition and inflammation in patients with ESRD.

## Conclusions

We have shown that the presence of higher MIA score before kidney transplantation is an independent predictor of post-transplant ACS. Further investigations are needed to assess whether improving MIA syndrome would impact the occurrence of ACS after kidney transplantation and whether active evaluation and treatment for coronary artery disease would be helpful for improving outcome in patients with high MIA score.

## Abbreviations

CVD: Cardiovascular disease
ESRD: end-stage renal disease
MIA: Malnutrition, inflammation, and atherosclerosis
GFR: glomerular filtration rate
MICS: malnutrition inflammation complex syndrome
NODAT: new onset diabetes after transplantation
CRP: C-reactive protein
BMI: body mass index
DM: diabetes mellitus
ACS: acute coronary syndrome
MI: myocardial infarction
HR: hazard ratio
CI: confidence interval
PTH: parathyroid hormone
CMV: cytomegalovirus
